# Language evolution and recursive thought

**DOI:** 10.3389/fpsyg.2013.00812

**Published:** 2013-10-30

**Authors:** Iain DeWitt

**Affiliations:** Laboratory of Integrative Neuroscience and Cognition, Department of Neuroscience, Georgetown University Medical CenterWashington, DC, USA

**Keywords:** language evolution, syntax, universal grammar, cognition, primate cognition

In simplest terms, language is the syntactic combination of concepts (semantics), which are mnemonically addressed with man-made sensory-based representations (word-forms). The evolution of language, therefore, is minimally the evolution of competency for learning the grammar and words of a given language. Apes have an ability to learn symbol-concept associations across several modalities, albeit an impoverished ability. No conclusive demonstration, however, exists for grammatical processing in non-human primates. Grammar, thus, appears to be the more recent and unique innovation. (Speech, also unique, is secondary as it relates to expression and as sign language is equally expressive.) Chomsky and colleagues famously proposed grammatical recursion is “recently evolved and unique to our species” and that it is the minimum characteristic of the faculty of language (Hauser et al., 2002; Chomsky, 2010). In *The Recursive Mind*, Michael Corballis disputes this, arguing recursion's incorporation into other cognitive domains antedates its incorporation into language. Further, he argues the language faculty evolved for communication, not cognition:

I part from Chomsky … in his view that thought itself is fundamentally linguistic. I argue instead that the modes of thought that made language possible were nonlinguistic, but were nonetheless possessed of recursive properties to which language adapted … This change of view provides the main stimulus for this book … [It] leads to a better understanding of how we … think … [and] a radically different perspective on language itself, as well as on how it evolved. (Corballis, under review)

In linguistics, the core application of recursion is phrase embedding. Chomsky posits an operation, *unbounded Merge*, that recursively merges words to create larger phrases. For example, given, “Jane said Janice thought June was tired and emotional,” merge would construct something like: {Jane, {said, {Janice, {thought, {June, {was, {tired and emotional}}}}}}}. In Chomsky's view, the evolution of unbounded Merge is the genesis of language:

Within some small group from which we are descended, a rewiring of the brain took place in some individual, call him Prometheus, yielding the operation of unbounded Merge, applying to concepts with intricate (and little understood) properties … Prometheus's language provides him with an infinite array of structured expressions. (Chomsky, 2010)

Part I of Corballis' thesis is the question of priority: Did recursion evolve first in a non-linguistic domain? In the archeological record, technological artifacts give evidence for dating the emergence of recursive thought. The acheulian industry (1.6 mya) required a hierarchical method of production, indicative of recursion (Vaesen, 2012; Barceló-Coblijn and Gomila, 2012; Holloway, 2012). While modern cranial volumes (1.2 mya) are more recent than ascheulian technology, the archeological record gives no definitive evidence of the linguistic status of early hominins. Corballis' argument therefore focuses on the behavior of primates, who clearly lack syntax, for evidence of recursion antedating language. Corballis offers mental time travel and theory of mind as examples of non-linguistic domains that possess recursion and which evolved prior to the split between the human lineage and the lineages of our primate cousins. Unfortunately for Corballis' argument, neither mental time travel nor theory of mind are well established in primates. Curiously, Corballis concedes the point: “Evidence … indicates that nonhuman animals, even chimpanzees, are essentially incapable of theory of mind, mental time travel, or language—or are at best capable of these capacities only sporadically, and only in rudimentary fashion (Corballis, under review).” Thus, while Corballis may conjecture recursion's prior evolution within non-linguistic domains, at present, there is no evidence for this. Another problem for Corballis' position is that, strictly speaking, Chomsky's hypothesis is not that recursion evolved first within the language faculty. Rather, his hypothesis is that the integration of recursion into the precursor language faculty completed the genesis of the language faculty. Chomsky explicitly ponders recursion's incorporation *via* either *de novo* establishment or annexation (Chomsky, 2010).

Part II of Corballis's thesis is the question of purpose: Was language initially selected for its communicative function? To Chomsky, grammar is language's essential quality. It enables the structured, hierarchical combination of concepts into statements. It is effectively, if not exactly, an engine for symbolic thought. This, Chomsky posits, was its principal adaptive value at genesis. Corballis' thesis implies language's principal value at genesis was the ability to transmit non-linguistic thoughts to others, thoughts already possessed of hierarchical structure.

Presuming a primarily communicative origin, primacy of recursion in non-linguistic domains, and the centrality of grammar to language, early language might have been combinatorial but basically non-hierarchical—either flatly non-hierarchical or at least not deeply hierarchical. It would have been limited in the complexity of its statements but still capable of complex expression. For instance, “June was tired. June was emotional. Janice thought it. Jane said.” Though less compact, less precise, and reliant on implicature, such expressions succeed, more or less, in conveying hierarchically structured concepts. Notably—and sometimes bitterly controversial among linguists (Bartlett, 2012)—Daniel Everett has reported existence of exactly such a language, Pirahã, the language of an Amazonian tribe (Everett, 2005, 2012). The recursive nature of Pirahã is somewhat moot as it is sufficient to reference the Gedankenexperiment: We can all imagine a system of communicative expression that does not have subordinate clauses and otherwise behaves as Everett would claim Pirahã does. Clumsy though it may be, such language would suffice to provide some additional survival value to its speakers (or signers).

Chomsky asserts *bounded* Merge is an unlikely constituent of the precursor language faculty for reason that “there is no empirical evidence … for such stipulation”—an apparent dismissal of Everett—“and no obvious rationale … since it is still necessary to assume that at some point unbounded Merge appears (Chomsky, 2010).” Neither rebuttal bears much weight, thus we may conclude both the bounded Merge and Prometheus scenarios are viable. Finally, we should ask if it is clear that Corballis means anything fundamentally different than recursive symbolic thought with his notion of non-linguistic recursive thought. If the objects of his non-linguistic thought are abstract representations, though not words—in that the concepts are not referenced by word-forms—that are combined by computations similar to syntax, at what point is the linguistic–non-linguistic distinction only one of emphasis?

## References

[B1] Barceló-Coblijn L. Gomila A. (2012). Evidence of recursion in tool use. Behav. Brain Res. 35, 219–220 10.1017/S0140525X11001865 22697279

[B2] Bartlett T (2012). Angry Words. The Chronicle of Higher Education. Available online at: http://chronicle.com/article/Researchers-Findings-in-the/131260/

[B3] Chomsky N. (2010). Some simple evo devo theses: how true might they be for language? in The Evolution of Human Language, eds Larson R. K. Deprez V. Yamakido H. (Cambridge: Cambridge University Press), 45–62 10.1017/CBO9780511817755.003

[B5] Everett D. L. (2005). Cultural constraints on grammar and cognition in Pirahã: another look at the design features of human language. Curr. Anthropol. 46, 621–646 10.1086/431525

[B6] Everett D. L. (2012). Language: The Cultural Tool. New York, NY: Pantheon Books

[B7] Hauser M. D. Chomsky N. Fitch W. T. (2002). The faculty of language: what is it, who has it, and how did it evolve? Science 298, 1569–1579 10.1126/science.298.5598.1569 12446899

[B8] Holloway R. L. (2012). Language and tool making are similar cognitive processes. Behav. Brain Res. 35, 226 10.1017/S0140525X11002019 22697167

[B9] Vaesen K. (2012). The cognitive bases of human tool use. Behav. Brain Sci. 35, 203–218 10.1017/S0140525X11001452 22697258

